# Research Progress and Trends on Utilization of Lignocellulosic Residues as Supports for Enzyme Immobilization via Advanced Bibliometric Analysis

**DOI:** 10.3390/polym15092057

**Published:** 2023-04-26

**Authors:** Francisco Simão Neto, Maria Marliete Fernandes de Melo Neta, Misael Bessa Sales, Francisco Arisson Silva de Oliveira, Viviane de Castro Bizerra, Ada Amélia Sanders Lopes, Maria Alexsandra de Sousa Rios, José Cleiton Sousa dos Santos

**Affiliations:** 1 Departamento de Engenharia Química, Universidade Federal do Ceará, Campus do Pici, Bloco 709, Fortaleza 60440-554, Brazil; 2 Departamento de Engenharia Mecânica, Universidade Federal do Ceará, Campus do Pici, Bloco 714, Fortaleza 60440-554, Brazil; 3 Instituto de Engenharias e Desenvolvimento Sustentável, Universidade da Integração Internacional da Lusofonia Afro-Brasileira, Campus das Auroras, Redenção 62790-970, Brazil

**Keywords:** enzymatic immobilization, lignocellulosic biomass, bibliometric analysis

## Abstract

Lignocellulosic biomasses are used in several applications, such as energy production, materials, and biofuels. These applications result in increased consumption and waste generation of these materials. However, alternative uses are being developed to solve the problem of waste generated in the industry. Thus, research is carried out to ensure the use of these biomasses as enzymatic support. These surveys can be accompanied using the advanced bibliometric analysis tool that can help determine the biomasses used and other perspectives on the subject. With this, the present work aims to carry out an advanced bibliometric analysis approaching the main studies related to the use of lignocellulosic biomass as an enzymatic support. This study will be carried out by highlighting the main countries/regions that carry out productions, research areas that involve the theme, and future trends in these areas. It was observed that there is a cooperation between China, USA, and India, where China holds 28.07% of publications in this area, being the country with the greatest impact in the area. Finally, it is possible to define that the use of these new supports is a trend in the field of biotechnology.

## 1. Introduction

Given the development of the industry, new materials had to be developed or studied to analyze the possibility of use or reuse [1,2]. In this context, the use of waste from the industry has been more desired, because as the industry increases its production, more waste is generated [3,4]. These wastes are destined for reuse or are disposed of incorrectly, causing negative effects on the environment [5,6,7]. Thus, the proper disposal of this waste has come into focus, and studies have been conducted to improve this area [8,9].

As research on reuse advanced, scientists concluded that agro-industrial wastes had the potential for reuse [10,11]. Within this class of waste are the residues of lignocellulosic materials, which have great potential for various lines of research [12,13,14]. These materials are represented by different residues, such as sugarcane bagasse [15]—used in ethanol production—cashew bagasse, and carnauba straw [16,17,18]. In the studies of potential applications for these materials, they saw the possibility of using them as a support for enzymes.

Enzymes are macromolecules that have the potential to catalyze many reactions [19,20,21,22,23,24].

There are different ways in which to obtain the enzymes from different microorganisms, resulting in the classification as a biocatalyst [25,26]. These biocatalysts have high selectivity and purity, thereby reducing the generation of byproducts in the reactions where they are applied [27,28,29]. However, these biocatalysts may suffer interference from the reaction medium, as they are susceptible to denaturation by organic solvents and damage to their catalytic site [30,31,32,33,34,35].

As enzymes demand very specific technology and, a growing industrial demand, these bioproducts have high market value [24,36,37,38,39,40,41,42,43,44,45,46,47]. Thus, a new area of research emerged, where studies have been carried out to enable enzyme immobilization [48,49,50,51,52]. The immobilization results in the potentiation of the catalytic capacities of these enzymes, improving the stability and resistance to denaturation in organic medium, facilitating the separation of the reaction medium, and the reuse of this biocatalyst [38,46,49,53,54,55,56,57,58,59,60,61,62,63]. In the studies, new materials are analyzed to be used as support, which resulted in the possibility of applying lignocellulosic materials [46,64,65].

We performed the analyses based on the advanced bibliometrics method [41,66]. This method involves a thorough review of all works that were published within the time defined in the method [67,68]. It resulted in the visualization of countries relevant to the topic in question, principal organizations involved in the research, journals that stand out in the publication of results in this area, and leading authors working on the topic. In addition, it resulted in the analysis of cooperation between the following spheres: country, organization, authors, and journals [41,69,70]. This analysis can also help predict the future of this research topic, even using data from previous years [71,72].

Thus, an advanced bibliometric analysis considered keywords, co-citation links, and bibliographic coupling networks as the main units of analysis to address the following research questions (RQs):

- RQ1. What are the advances in scientific production on lignocellulosic biomass and enzymatic immobilization?
- RQ2. Research hotspots (keywords) that stand out in studies of lignocellulosic biomass and enzymatic immobilization?
- RQ3. Which authors, organizations, and countries stand out in studies on lignocellulosic biomass and enzymatic immobilization?
- RQ4. What are the main emerging research subfields of lignocellulosic biomass and enzyme immobilization in recent literature?

**Figure 1:**
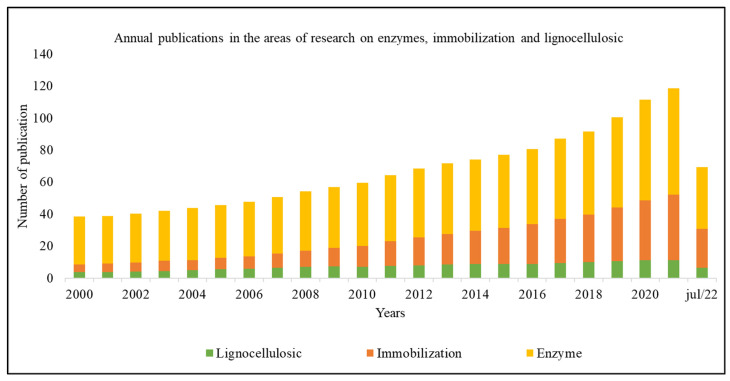
Annual production of research on enzymatic immobilization using lignocellulosic materials as support.

As shown in Figure 1, there has been an increase in publications involving enzymatic immobilization. Such research is mainly motivated by environmental issues, which have recently been gaining prominence in scientific discussions. Thus, the year 2021 can be highlighted with the number of publications related to enzymatic immobilization in lignocellulosic materials, proportionally higher compared to previous years, with about 118 academic productions. Then there is the year 2020, with 112 academic productions, and then the year 2019, with about 112 academic productions. The productions for the year 2022 are less expressive since only the data from January to July were considered the time of construction of the database, however, even in this reduced period, about 70 articles had already been published. that there has been a growing interest in the scientific community in relation to this area of research.

Still, in relation to Figure 1, it is possible to verify that, separately, the themes of immobilization and enzymes were more addressed in the year 2021, with 41 and 66 articles being published this year, respectively. On the other hand, the topic of biomass and lignocellulosic materials was addressed more frequently in 2020, with a total of 11 articles.

Thus, the present work aims to perform an advanced bibliometric analysis of the applications of lignocellulosic materials as support for enzyme immobilization. It is justified by the need for new enzyme support materials and by developing new applications for agro-industrial waste, thereby reducing the environmental impacts involved in the improper disposal of this waste.

## 2. Methodology

### 2.1. Data Source

The data used in the paper were acquired from the Web of Science—Core Collection website database. For data selection, classification parameters referring to advanced bibliometric analysis were applied. In accessing the platform, the login is provided by the Coordination for the Improvement of Higher Education Personnel (CAPES). The access was provided through the Periódicos Capes website.

**Figure 2:**
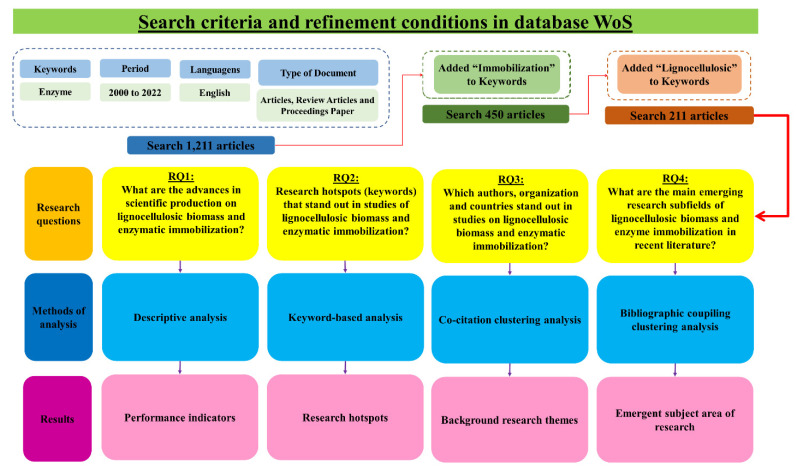
The structure that represents the search and analysis criteria.

Figure 2 shows how the acquisition of the data that comprises the primary database was performed. This database will support all the analyses that will be performed during the work. The term “Enzymes” was inserted in the research line, and we delimited the filter as “Title”. Then a new row was inserted with the term “2000–2022” and the filter “Year of Publication”. One thousand two hundred and fifty-eight articles were retrieved. These were the articles that fell within the limits of the research; however, to improve the analysis, refinement was necessary to ensure the data fidelity, so discarding the academic papers that escaped the purpose of this bibliometric analysis.

The delimitations were performed in the following way: Document Types, where the limitation was restricted to Articles, Conference Papers, and Review Articles; Language, where only English language works, would be considered. Finally, the dataset used was limited to 1211 articles that will be applied in the advanced bibliometric analysis method. Subsequently, the primary database resulted in generating two new smaller databases that would be used to analyze two other topics relevant to the research topic.

The first derivation led to analyzing the feedstocks used for enzyme immobilization. A new line was added to delimit a new term: the filter used remained as “All Fields”, the term was defined as “biomass” or “lignocellulosic”, and the other filters and terms were maintained to give continuity to the 1211 articles already defined. After this new delimitation, we found 255 articles related to the searched terms (i.e., the use of biomass and lignocellulosic materials in works that involve enzymes) from 2000 to 2022.

The second derivation applied concerns the treatment performed on the enzymes, to ensure greater resistance and thus guarantee greater possibilities for application under different conditions. This treatment is immobilization. For this reason, the new line used the term “Immobilization” with the filter “All Fields”. Thus, a return of 450 articles related to the searched topic was obtained, again within the 1211 articles in the database.

### 2.2. Data Analysis

Three software programs were used for analysis in this work: CiteSpace (version 5.8.R6, Philadelphia, PA, USA), VOSviewer (version 1.6.17 Leiden, South Holland, The Netherlands), and ArcGIS (version 10.5, Redlands, CA, USA). In order of application, in VOSviewer, data visualization was performed to allow a better understanding and analysis of the data [73]. Afterward, ArcGIS 10.5 was applied to present data distributions on geographic maps. Finally, CiteSpace was used to project the future of research in areas of interest based on keywords and generated clusters [74].

## 3. Results and Discussion

### 3.1. Enzymatic Immobilization

**Figure 3:**
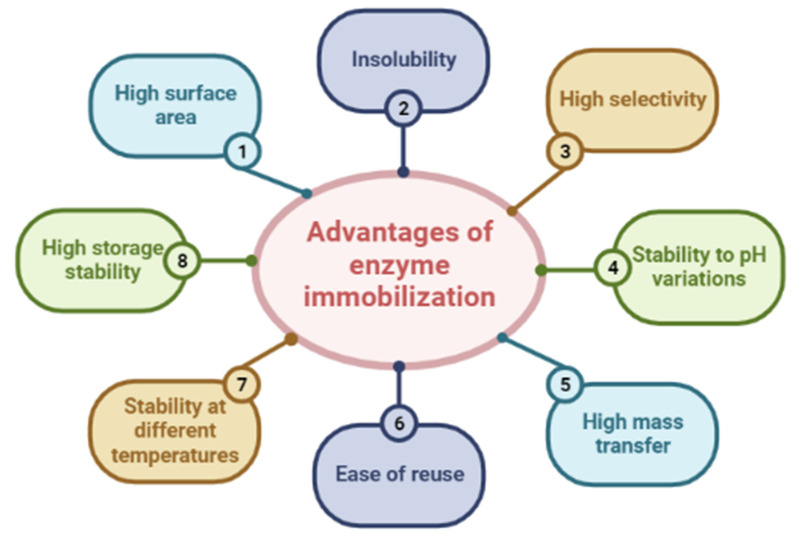
Advantages associated with enzymatic immobilization.

Enzymes are biocatalysts that can be applied in different situations [38,58,75,76,77,78]. This versatility makes the enzymes undergo modifications that guarantee the necessary resistance for each application [29]. Thus, enzymatic immobilization can optimize these biocatalysts, allowing their use in the most diverse research areas [45,79,80,81,82,83,84,85,86,87,88]. In Figure 3, it is possible to observe the advantages of this immobilization process, which may be responsible for several improvements in its stability in the reaction medium, increased resistance to different temperatures, pH, and organic solvents, and increasing its storage capacity and even allowing its reuse.

**Figure 4:**
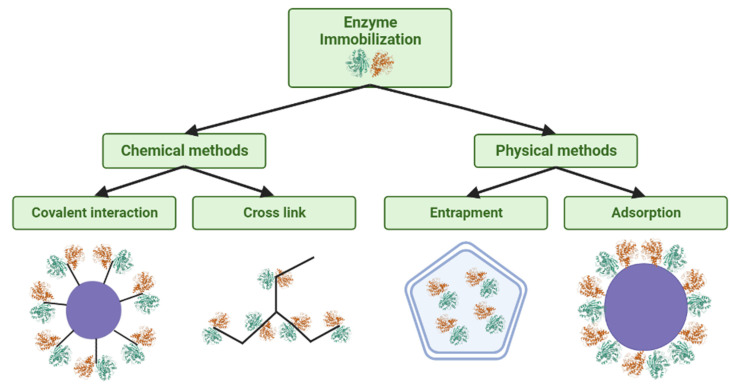
Main methods of enzymatic immobilization.

The enzymatic immobilization process can occur by different methods, whether physical or chemical [37]. The most used methods are divided into entrapment, where the enzyme is immobilized within an inert support, by binding directly to the support, where this method is divided into physical adsorption and binding or covalent coupling, and finally, cross-linking, which is the mutual binding between enzymes or proteins that are not soluble [36]. All methods have their associated advantages and disadvantages, so it is up to the researcher to analyze the characteristics of their application to define the most appropriate immobilization method [47]. Figure 4 exemplifies the biocatalysts obtained in each method.

The immobilization method by entrapment is within the classification of a physical method in which the enzyme is trapped inside the chosen support. In addition, it should be noted that this immobilization becomes irreversible in the case of a physical method [89,90]. Immobilization by entrapment has its methodology based on the polymer chain of the support, which after activation, starts to react with the enzymes, resulting in the formation of a polymeric matrix that will become a trapping structure for these biocatalysts [90,91]. Like other immobilization methods, entrapment allows more excellent resistance to pH variation, control of polarity and affinity, and other properties of this material to increase the catalytic power of these biocatalysts [92,93]. Regarding the materials used in immobilization by entrapment, it is possible to highlight collagen, gelatin, polyurethane, and alginate [94].

Enzymatic immobilization by physical adsorption was one of the pioneering methods in this line of research [95]. This easy-to-perform stabilization method can be reversible, occurring from hydrophobic interactions, hydrogen bonds, and van der Waals forces. [96,97]. The advantages of enzymatic immobilization by adsorption are the simplicity of the technique, which allows stabilization under mild conditions, the potential for maintaining the catalytic activity of the enzyme due to the absence of significant structural changes in the biomolecules, and the support that favors reuse [64,98]. However, the disadvantage is the stochastic nature of the enzymatic reaction and possible desorption due to changes in temperature, pH, and ionic strength [99,100]. Thus, alternative ways of overcoming these disadvantages have resulted in the development of several in recent years, such as the chemical modification of support materials and the use of crosslinking agents [101,102].

Covalent binding is one of the proteomic research’s most widely used enzymatic stabilization methods. It is based on covalent interactions involving the functional groups on the surface of the supports and amino acid residues of the enzyme [44,85]. In this immobilization method, spacer reagents are often used, whose purpose is to allow more significant contact of the enzyme with the reaction medium, where the real need for this will depend on the volume of functional groups added to the support [103,104]. As an advantage of this method, the more excellent resistance to variation in pH, temperature, and organic solvents stands out [105].

The cross-linking technique is an irreversible immobilization and does not require support [106,107]. In this method, the binding is mutual between the enzymes, but bindings with inactive proteins can also occur, thus forming complex three-dimensional structures [108]. Cross-linking forms an intermolecular bond involving the enzyme and the bifunctional or multifunctional reagent, making them insoluble in the reaction medium [109]. Binding with specific amino acid groups of the enzymes is only possible due to the presence of at least two interacting ends in the molecules that are the cross-linking agents [110]. Glutaraldehyde stands out as one of the most used reagents in this technique due to its low cost and availability [111]. This technique is laborious and requires much time, in addition to the potential loss of half of the enzymatic activity, reduced mechanical stability, and low reproducibility, especially when working with many enzymes [13,112].

### 3.2. Supports

The properties of inactivated enzymes are subject to the specific properties of the carrier and the enzyme, as well as the conditions of use of the carrier biomolecule [113]. The relationship between the two provides kinetic properties that are crucial for their practical applications [114]. Therefore, the conformational structure and the specific activity of the enzyme must be preserved when the immobilization is performed without the enzyme being defunctionalized when it comes into contact with the surface of the support [115].

Thus, carefully selected support can significantly enhance the performance of the system’s operation [60]. In terms of morphology, solid supports can be divided into porous and non-porous [116]. In terms of chemical composition, solid supports can be divided into organic and inorganic materials [117].

#### 3.2.1. Agroindustrial Waste

Agricultural and industrial waste is generally the by-products from manufacturing processes and industrial treatment of agricultural or animal products [118]. Straw, logs, bark, vegetable pulp, legumes, and many others are by-products of industrial processes that do not have added value, as these residues are often not used directly [119]. However, sugars, proteins, fibers, and minerals are compounds highly present in the composition of these by-products, thus providing alternative sources of carbohydrates and nitrogen to the detriment of synthesizing sources of these nutrients applied in bioprocesses [120].

Agribusiness and agricultural residues are alternatives for supplementing ruminant animals in critical periods when the availability of dry food decreases due to the lower availability of forage [121]. Residues such as corn straw, bean pods, husks, and straw from cereals such as rice, carnauba straw, and wheat straw have reduced nutritional value, despite their high availability [122]. These by-products still have a high lignin content, reduced soluble carbohydrate content, and a percentage of crude protein below the necessary [123].

The search for effective utilization of various agricultural and industrial wastes has increased [124]. Numerous bioprocesses have been designed to make it possible to use these materials as substrates in synthesizing multiple molecules with high-added value, such as microbial proteins, organic acids, ethanol, and enzymes [121]. Using agro-industrial by-products as a substrate for bioprocesses not only makes economic sense but also helps solve the environmental problems arising from accumulating these materials in the environment [120].

#### 3.2.2. Lignocellulosic Materials

**Figure 5:**
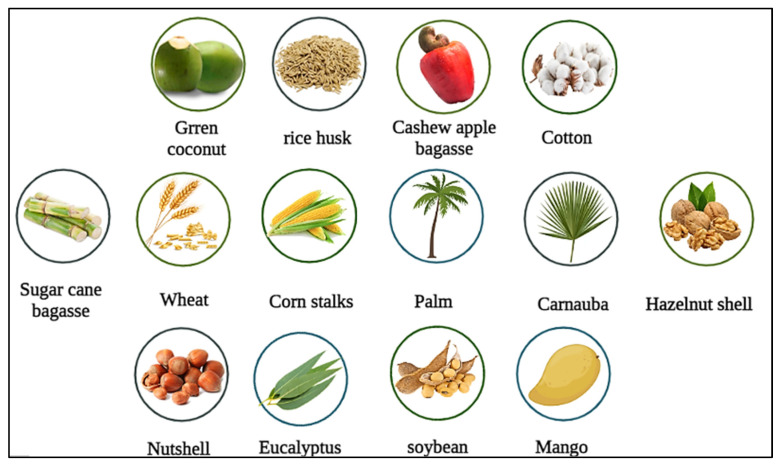
Examples of Lignocellulosic biomass for enzymatic immobilization.

It is estimated that 1.5 trillion tons of lignocellulosic material are produced annually, providing a renewable source of raw materials for synthesizing bioproducts such as ethanol and biodiesel [12]. This vast collection of lignocellulosic materials is mainly due to the management of crops [11]. In this context, it is essential to highlight a large amount of solid biomass that remains unused after harvesting seeds, grains, and residues from industrial production [14]. Figure 5 presents a series of lignocellulosic biomasses applied in the stabilization of enzymes.

Brazil is responsible for growing a wide variety of grains and crops, resulting in high amounts of agro-industrial waste that could be converted into ethanol. Worldwide, Brazil has produced 95 million tons of soy, placing it in second place in the production of this legume. In addition, it is also responsible for the production of 12 million tons of rice, placing it among the ten countries that produce more of this cereal.

The composition, molecular structure, and physical-chemical properties of lignocellulosic materials give them remarkable resistance to hydrolysis reactions [125]. Such a characteristic (resilience to hydrolysis) made it challenging to produce knowledge about economic techniques used to convert lignocellulosic materials into fermentable, energetically viable, and environmentally correct sugars [126].

Lignocellulose is an essential part of plants’ composition; it is directly linked to its structural composition and is a renewable material [127]. This lignocellulosic material is formed by three main components, cellulose (up to 50%), hemicellulose (20 to 40%), and lignin (20 to 30%), but it is possible to find extracts and inorganic compounds included in the material under analysis [128,129]. Each plant has its composition, and, in addition, factors such as age, harvest time, and state or stage of development also affect this composition [130]. The three most common polymers in structures are cellulose, hemicellulose, and lignin, which are highly entangled and chemically linked by variable bonds between non-covalent and cross-covalent [131].

Cellulose is a semicrystalline linear polymer formed from D-glucose subunits linked by β-1 glycosidic bonds to form cellobiose dimers [132]. Here, there is the formation of long elemental chains that are held together by hydrogen bonds and van der Waals forces [133]. Cellulose is commonly found in crystalline form compared to amorphous form [134].

Hemicellulose is a polysaccharide with a lower molecular weight than cellulose [135]. It is composed of D-xylose, D-mannose, D-galactose, D-glucose, L-arabinose, -O-methyl- glucuronic acid, D-galacturonic acid, and D-glucuronic acid [126] Polysaccharides are linked by β-1, and sometimes β-1,3 glycosidic bonds [128]. Hemicellulose differs from cellulose in that it has filaments with short side chains made of numerous and different sugars, while cellulose consists of oligomers very susceptible to hydrolysis [127].

Finally, there is lignin, which binds to hemicellulose and cellulose and forms a physical barrier impermeable to plant cell walls [125]. It is the composition of cell walls to provide structural support, prevents permeability, ensures resistance to attack by microorganisms, and provides cell wall protection against oxidative stress [126]. It is an amorphous, water-insoluble, and optically inactive macromolecule composed of phenylpropane molecules coupled with on-hydrolyzable bonds [129].

### 3.3. Bibliometric Analysis

#### 3.3.1. Publication Result: Overall Results

We got 1211 records from the Web of Science, published between 2000 and 2022. One publication from May 2020 had the highest visibility and relevance to the present study. This paper is entitled “Immobilization of enzymes and cells in lignocellulosic materials” by Rodriguez-Restrepo, YA [11], regarding using lignocellulosic materials for enzyme immobilization, aiming at their application in fermentation, remediation of contaminated water and soil, solvent synthesis, and fine chemistry. This paper presents an alternative application of these materials, as their high production and improper disposal can cause environmental damage [11].

Lignocellulosic materials are mostly biomass residues that have been used in different applications, such as energy production, either thermal or electrical energy, materials, and biofuels, such as sugarcane bagasse after bioethanol production [136]. The numerous applications of these biomasses increase their consumption and consequently, the production of waste of these materials, so alternative uses are developed to circumvent this problem. Currently, the application of these biomasses as enzyme support has gained considerable research prominence, as green coconut fibers [137], corn cob powder [14], and wood sawdust are already used [138] for covalent bond immobilization of *Candida antarctica* lipase, *Candida rugosa* lipase, among others [11].

Improving and modernizing the production of lignocellulosic materials is a decisive step in the production lines that produce these materials. Such changes will favorably influence the economics and results of by-product production, besides defining the ideal parameters for the growth of its use, thereby causing a decrease in the incorrect disposal of the by-products. The topics addressed by the articles analyzed in the database are used to highlight moments of great importance for environmental concerns and then show how to circumvent such setbacks to ensure a more sustainable lignocellulosic materials market.

#### 3.3.2. Distribution of Scientific Journals

After analyzing the database collected, it was possible to highlight the eligible works that are part of the theme of lignocellulosic materials as support for enzyme immobilization in the distribution of scientific journals. Thus, we found 391 journals, with an average of approximately 3.2 publications per journal. This result underlines the importance of this topic for several research areas. However, there is still a need to clarify this research line because of its excessive importance for scientific and industrial society. Another interesting point worth highlighting concerns the diversity of research groups working in this area, which reflects the wide variety of journals that address the topic. In addition, the different groups ensure the experimentation of many methodologies and individuals in each group. Such plurality guarantees a better explanation of the theme and enriches the academic community with new ideas and projects.

Table 1 summarizes the list of the ten journals with the highest influence on the theme of this paper, ranked by the number of publications. It is important to note that these journals hold more than a quarter of all publications analyzed here, which are not so expressive and reinforce the diversity of research in the area.

The journal Bioresource Technology stands out by leading the list presented in Table 1. This journal has an impact factor of 11,889 and 51 articles on this research topic, which represents 4.21% of the total papers collected. Further, it got 2913 citations over the years. Bioresource Technology obtained an average of 57.12 citations per article. The journal that ranks second on the list—Applied Biochemistry and Biotechnology—which has an impact factor of 3094, has 50 articles published on the topic, which represents 4.13% of the documents analyzed in this paper, and has 747 citations.

The list presented in Table 1 is mostly composed of European journals. Only Applied Biochemistry and Biotechnology and Enzyme and Microbial Technology are American journals. Although none of them is the journal with the highest impact factor, they are among the top ten selected. These scientific journals rank second and third, with the highest number of publications. This shows the excessive density of journals in the same region with regard to the topics studied here, as about 70% of the major journals are European.

The journals occupying the first and second positions in the ranking of Table 1 (Bioresource Technology and Applied Biochemistry and Biotechnology) hold 30% of the most cited articles related to enzyme immobilization. Thus, one can see that there is a large distribution of studies carried out from a methodology of specific scientific groups. All the 10 most cited journals together account for only 23% of all publications in the database collected, among the 391 journals analyzed. Regarding the number of citations, Bioresource Technology has 2913 (9.71% of the total citations) and Applied Biochemistry and Biotechnology has 1774 (5.71% of the citations). This shows the relevance of publications from these journals and the wide dispersion of studies with less prominence in a wide variety of journals.

#### 3.3.3. Distribution by Countries, Organizations, and Authors

We analyzed the relationship between articles and their countries and institutions. Among the 81 countries cited in the manuscripts, 10 countries concentrated most of the publications—about 87.81% of the papers. The remaining articles are from the other 71 countries. Table 2 shows the leading countries.

There is a greater interest in this topic from some central regions, displayed by the concentration of articles in a smaller portion of the stated countries. China holds 28.07% of the articles published on the topic, thereby securing its position at the top of this list. India holds 13.46% of production—half that seen in the country in the first place. Finally, in third place is the United States of America, with 10.4% of production, a figure close to second place that leaves it even further away from first place.

**Figure 6:**
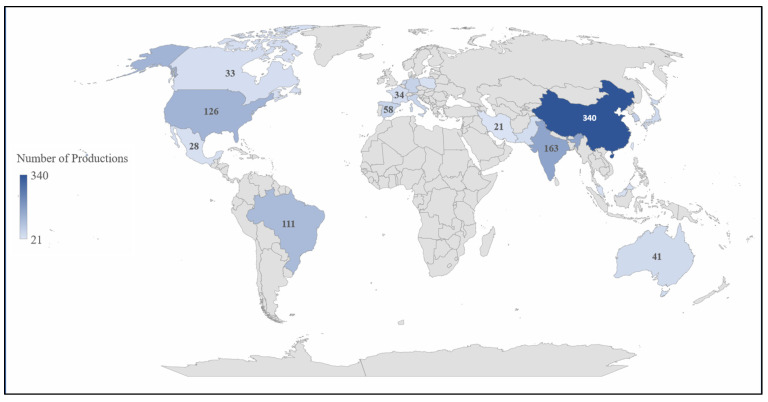
Representation of the distribution of articles by country.

The following analysis considers the number of citations per country to determine the relevance and impact of these countries. Once again, China holds 7419 citations, which places it as the country with the highest impact regarding lignocellulosic materials as a support for enzyme immobilization. India was the second country with the most citations (3323 citations), followed by the United States of America (2555). The number of citations is directly linked to the number of manuscripts produced, as these countries are the largest holders of articles (Figure 6). For the construction of this figure, 19 regions that published at least 20 articles were specified.

**Figure 7:**
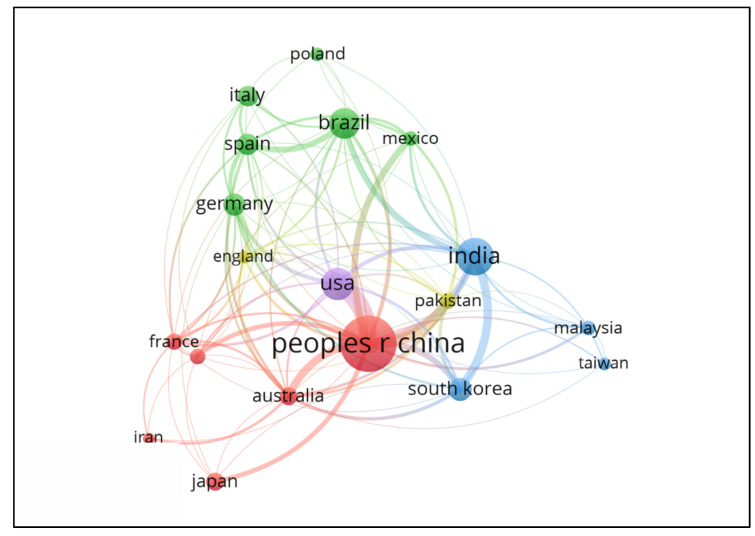
Network visualization map related to collaboration among the 19 countries with the most publications. The thickness of the lines connecting the two countries indicates the accumulation of co-authorship, and the colors divided into clusters illustrate the groups of countries with a high level of collaboration.

Considering the filters used in Figure 6, Figure 7 represents the network of interconnections or the collaboration network between countries. The great interaction between China and the United States is clear, ensuring a good relationship between sharing ideas and working together to build manuscripts, although these countries are on different continents.

The economic factor is also responsible for bringing these countries together since they are world economic powers, and scientific cooperation strengthens both. In addition, there is a concern with environmental problems, a problem that these countries deal with regularly. Thus, cooperation guarantees investments in this area and the possibility of exchanging ideas. Figure 1 shows this point, which represents the growing interest in studies regarding lignocellulosic materials as a support for enzyme immobilization. Finally, the interconnections show a triangle of cooperation between China, India, and South Korea, valid because of their geographical proximity, showing the proximity between their economic and socio-environmental interests.

After analyzing the database, we noticed the involvement of 1482 organizations from 81 countries. This expressive number testifies to the importance of the present work. However, the greatest volume of manuscripts produced is focused on about 20 countries, which together represent only a quarter of all the countries identified. Emerging and developed economies are related to developing alternative solutions that guarantee a good socio-environmental policy, which is in line with the countries highlighted in this analysis: China, India, the USA, and Brazil.

We found that 98% of the organizations cited produced up to 10 publications on this research topic from 2000 to 2022. About 70% produced only one publication. We could verify the contribution of several authors that ensured the research decentralization, even within the same country, thanks to this perspective introduced by the analysis. However, consistency in the production of manuscripts in this area is restricted to only 2% of the organizations.

Two organizations that each have only one manuscript on the topic have a high number of citations. The first is Meisei University (Japan) which owns the manuscript “Ethanol fermentation from biomass resources: current status and prospects” by Lin, Y [139]—with 1118 citations, which makes it the most cited article in the database. The second is Lund University (Sweden) which has the article “Fermentation of lignocellulosic hydrolysates. I: inhibition and detoxification” by Palmqvist, E [10]—with 939 citations, ranking second in the ranking of most cited articles.

The data analyzed show strong links between countries geographically close, which is valid because of cooperation between neighboring organizations. However, some countries have organizations that break out of this geographical barrier and form partnerships with organizations from other continents, such as the partnership between the United States and China. Brazil also stands out in the interconnection of organizations on different continents, having a close relationship with Spain and India. The relationship between Brazil and India is strong in the pharmaceutical market, which may justify academic cooperation between these two countries [140].

**Figure 8:**
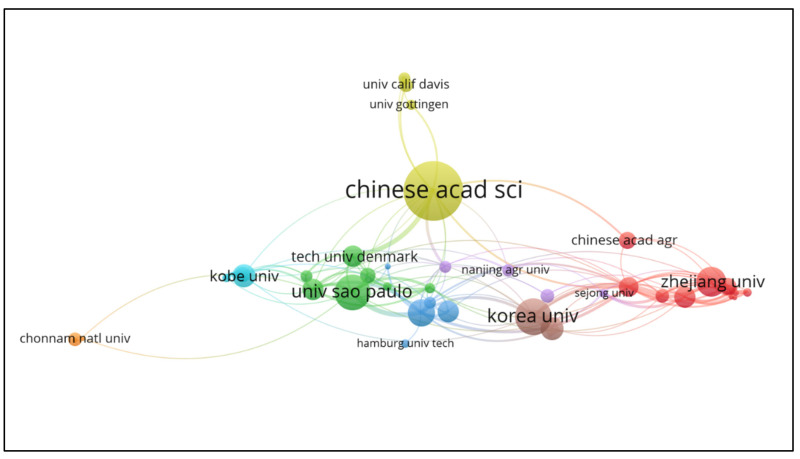
Network visualization map related to collaboration between organizations with at least 250 citations. The thickness of the lines connecting the two organizations is a strong indication of the accumulation of co-authorship, and the colors divided into clusters illustrate groups of institutions with a high level of collaboration.

Figure 8 illustrates the interconnections between the organizations. For a proper presentation of the interactions, we delimited a minimum amount of 250 citations accumulated by documents published in the analyzed period, thus identifying 40 institutions in the database that meet the minimum citation quota. We highlighted the most relevant organizations, such as the Chinese Academy of Sciences, the University of São Paulo, and the University of Korea, with the best cooperation results. Finally, among the 47 institutions with at least 250 cumulative citations, only 21 (about 44.7%) had over 10 collaborations with other organizations.

**Figure 9:**
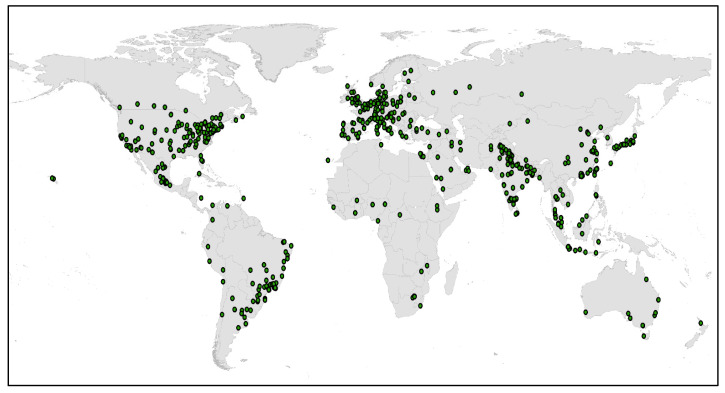
Geocoding of organizations registered in the 1211 articles analyzed.

To complement the network visualization analysis, a geocoding was developed that represents each organization within its country as green dots. This geocoding increases the possibilities of visualization and analysis of the organizational distribution involved in the research focused on the research topic. Figure 9 presents an option for analyzing geocoded addresses, showing a higher density of organizations in the regions that comprise North America and Europe, and not far below some regions of South America and Asia.

**Figure 10:**
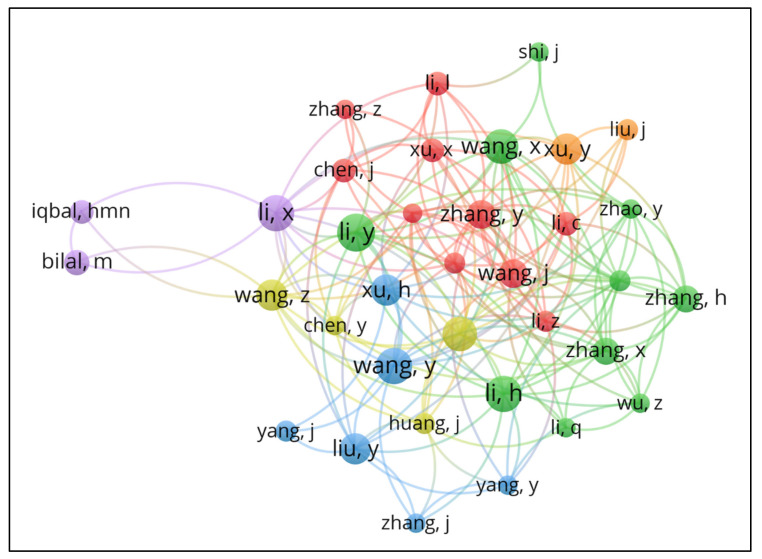
Network visualization map related to collaboration between authors with at least 450 citations. The thickness of the lines connecting two authors is a strong indication of the accumulation of co-authorships, and the colors divided into clusters illustrate groups of authors with a high level of collaboration.

A total of 5323 different authors were identified. This result shows the great diffusion of research lines carried out in this area, which guarantees a great diversity of researchers and methodologies to apply to the theme. Figure 10 presents the relationship between authors, following the delimitation that each author must have at least one published work and have at least 450 citations of their works. This image provides a view of the closest collaboration sets and shows that almost all authors are part of the large collaboration network for co-authorship.

The author Lin, Y authored the most cited paper in the database (“Ethanol ferment from biomass resources: current state and prospects” [139]), with 1018 citations. However, we notice he is not part of an extensive network of collaborations with other papers. On the other hand, Kim, Seung participated in developing 14 papers (most notably “Enzymatic coproduction of biodiesel and glycerol carbonate from soybean oil and dimethyl carbonate”, from 2011), which obtained 69 citations [141,142,143,144,145,146,147,148,149,150,151,152,153,154].

We implemented a filter of at least five citations that reduced authors to 3810, of which only 10 had no co-authorship relationship. These remained outside the primary network, generating ten smaller and independent networks, which represent about 0.26% of the collaborating authors. Thus, it is once again clear the large collaborative network for the production of articles, the local networks between neighboring institutions, and the cooperation between authors from the same institution, which in the end all collaborations strengthen the studies on this theme.

#### 3.3.4. The Most Cited Articles

From the analysis of the most cited manuscripts about lignocellulosic materials as support for enzyme immobilization, 10 articles stood out, which together obtained 5753 citations. The manuscript “Ethanol fermentation from biomass resources: current state and prospects” [139], which is in the first place, obtained 1018 citations, thus accounting for 17.7% of the total citations (Table 3). This article addresses ethanol fermentation, presenting practical examples and providing information for a general and rather broad overview of the current ethanol fermentation scenario. Above all, it cites the biomass resources, microorganisms, and technology applied in this production. In addition, it presents promising perspectives for ethanol fermentation.

The paper “Fermentation of lignocellulosic hydrolysates. I: inhibition and detoxification” [10] obtained 939 citations in the analyzed time interval. This manuscript presents a review regarding the effect of different detoxification methods on fermentability and the chemical composition of hydrolysis components. Thus, the inhibition of fermentation is mitigated by the application of a ligninolytic enzyme. lacase, by prior fermentation with the fungus Trichoderma reesei and other pretreatments. Another point analyzed is the yield of the fermentative reactions, identifying possible inhibitors linked to continuous and discontinuous fermentations [10].

Another prominent paper is entitled “Fungus-mediated synthesis of silver nanoparticles and their immobilization in the mycelial matrix: a novel biological approach for nanoparticle synthesis” [155], which is about a pioneering biological method applied to the synthesis of silver nanoparticles with Verticillium. By leaving the fungal-based biomass in contact with aqueous Ag+ ions, intracellular metal ions and the formation of silver nanoparticles of 25 ± 12 nm were reduced. Electron microscopic analysis of fungal cell flakes showed that Ag particles formed under the cell wall, probably because of metal ions from enzymes in the cell wall membrane. Finally, it was concluded that metal ions showed no toxicity to fungal cells and could continue their proliferation after the biological synthesis of Ag nanoparticles [155].

The article “White rot fungi and their enzymes to treat industrial dye effluents” [156], also stands out as it is a review of the future uses of white rot fungi (WRF) and their embryo-modifying enzymes (LMEs) in treating industrial effluents, especially those contaminated with dyes. The textile industry is by far the most enthusiastic user of synthetic dyes and demands environmentally friendly solutions for colored wastewater. The potential for bleaching and reducing WRF intoxication can be exploited with new knowledge about the physiology of these organisms, their bio-stimulatory properties, and the stability of their enzymes. This knowledge should be translated into robust and reliable waste processing [156].

#### 3.3.5. The Research Areas

**Figure 11:**
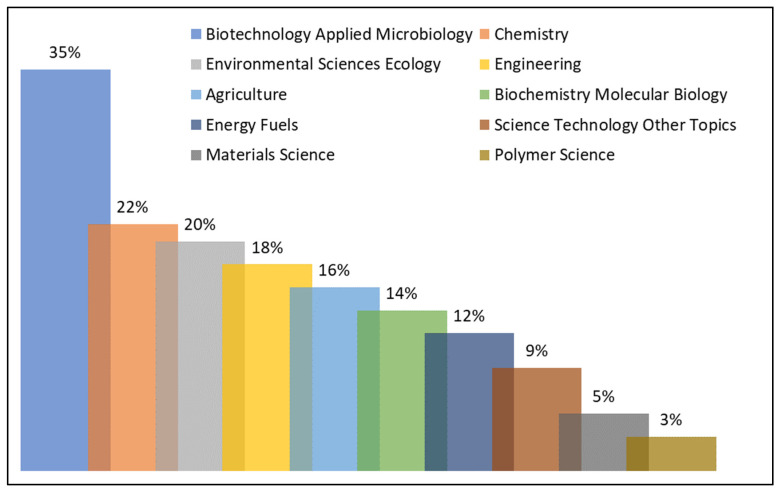
Distribution of research areas related to lignocellulosic materials as support for enzymatic immobilization.

After searching the database, we found 40 research areas on lignocellulosic materials as support for enzyme immobilization among the 1211 articles published from 2000 to 2022. We highlight the principal research lines in Figure 11. The journal Biotechnology Applied to Microbiology stood out the most, with 426 occurrences (23% of the manuscripts). In the sequence, the area of Chemistry obtained 262 records, occupying the second place in this category and representing 14% of the total articles analyzed.

It is possible to verify that research is not centralized in a few areas, but well distributed in several others. Such distribution is favorable because it ensures greater applicability of immobilized enzymes and increased demand for this material. As a reflection of the demand for a certain material, it reduces its market value because of the supply and demand theory. Another reflection of the large-scale use of these immobilized enzymes ensures a significant reduction in the impacts caused to the environment by conventional chemical processes.

Finally, we must clarify that there was a delimitation in the database, excluding some manuscripts that were not relevant to the topic of this study. Therefore, only papers that fell into the category of article, review article, and conference paper were considered in this analysis.

### 3.4. Hot Research Topics

#### 3.4.1. Quantitative Analysis of Frequent Keywords

The analysis of the keywords determines the amount of research developed in certain areas, thus projecting the growth trends of discussions in these areas and which parameters are followed by the authors. This information is necessary because together it confirms the development of the area studied. Table 4 ranks the 20 most relevant keywords among the manuscripts present in the database of this study. Another point addressed in this table is the strength of the link that each word has.

**Figure 12:**
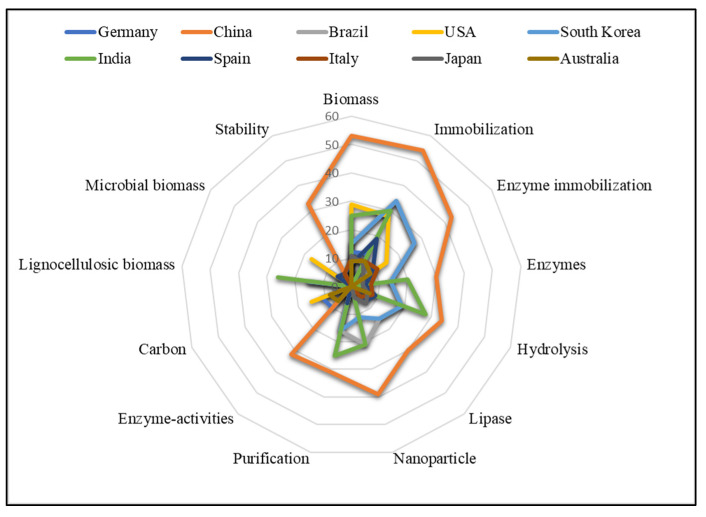
Keyword radar map of top 10 countries from 2000 to 2022.

Furthermore, from the keyword analysis, in Figure 12 of the radar map, the difference in the search direction of the top 10 producing countries. Studies in the United States, China, Brazil, Spain, Australia, India, South Korea, Italy, and Germany show different topics in this field.

**Figure 13:**
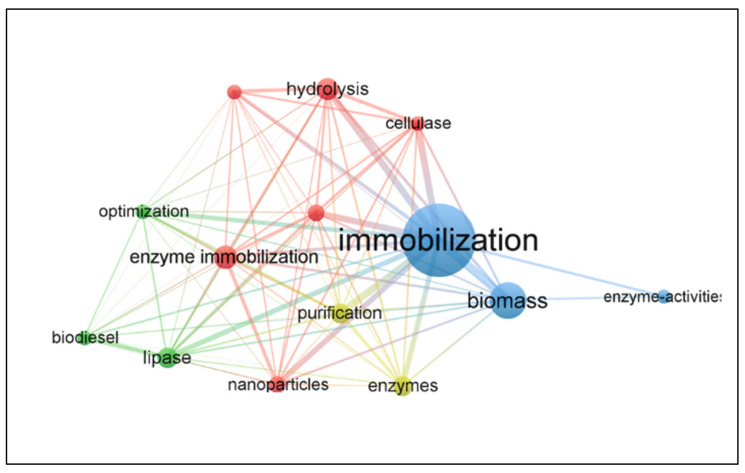
Keyword visualization network from January 2000 to December 2022.

Figure 13 shows the relationship and application trend of the main keywords present in the database. This analysis allowed us to conclude that in the period from 2000 to 2022, the word that had the greatest impact was “immobilization” (385 occurrences). The result of this highlighted word represents the direction of research for this area. In addition, other prominent words follow the same application logic (i.e., are linked to immobilization), such as the keywords “biomass” and “immobilized enzyme”, which have 192 and 116 occurrences in this connection network, respectively. The size of the links represent the relationships between words; therefore, the larger the link, the greater the interaction between them.

**Figure 14:**
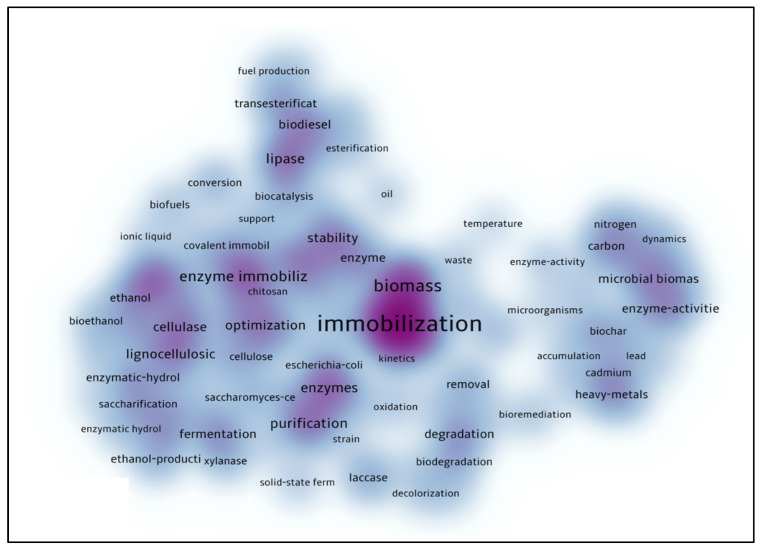
Co-citation density map of keywords in the database related to lignocellulosic materials as support for enzymatic immobilization.

**Figure 15:**
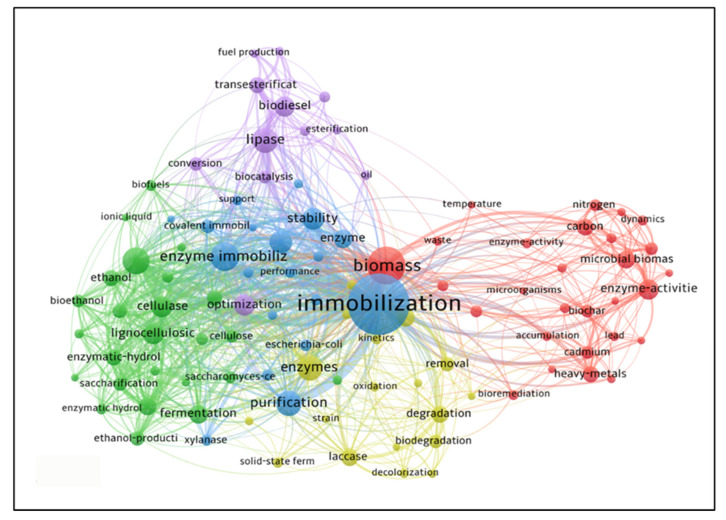
Visualization map of keyword co-citation network in research related to lignocellulosic materials as support for enzyme immobilization.

Figure 14 shows the keyword density map, points with more intense colors show keywords with a higher number of occurrences. The delimitation for this network was at least 20 occurrences in different articles, which resulted in a set of 100 words. In addition, Figure 15 results from another analysis of the keywords, by which it was possible to obtain a broader network of the use of these words. However, in Figure 15, they relate to a wider range of areas represented by the words. The larger number of items in the network allows the generation of islands of words that are part of the same search field, where, in the image, a different color represents each field. This image follows the same criteria used previously.

The cluster symbolized by the blue color is the most relevant because it contains the most frequent keyword—“Immobilization”—which is directly in line with the theme of this study. Still in this cluster are the keywords “enzyme immobilization”, “lipase”, and “purification”, which together will represent a research field. The keyword “biomass” is the highlight of the red cluster and thus adds relevance to this group since its percentage of occurrence is perceived in the second position of the keywords ranking. The words “waste” and “microbial biomass” are highlights that are linked to “biomass” and thus define another research field.

The keyword “optimization” links the green and purple clusters, highlighted among the 20 most cited. However, these clusters differ in their application. The green cluster is linked to the keyword “fermentation”, which relates to “ethanol” and “bioethanol”, forming one research field. The purple cluster has “lipase” as a highlight, relating strongly to “biodiesel” and “esterification”, resulting in another distinct research field.

The yellow cluster focuses on issues related to the environment, using the keyword “enzyme” as a highlight and relating it to the keywords “biodegradation” and “removal”. These words are linked to the purification and selectivity promoted by enzymes, where such characteristics reduce the environmental impacts associated with conventional production routes [87].

**Figure 16:**
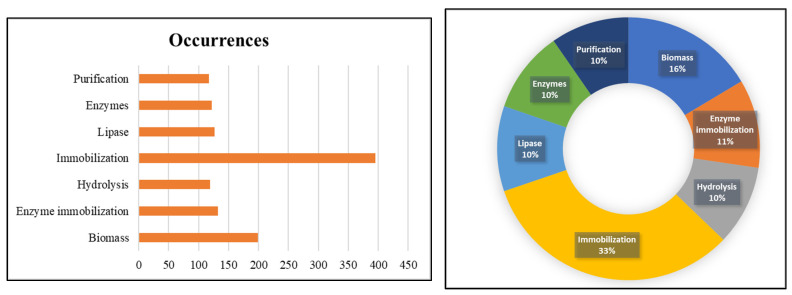
List of most cited keywords (**left**) and the percentage of articles on the occurrence of keywords (**right**) presented in the titles of the analyzed documents.

Finally, Figure 16 will demonstrate the occurrence relationship between some of the keywords used in works involving lignocellulosic materials, which resulted in the analysis of 1211 publications. By analyzing this graph, it is clear that Immobilization and Biomass, as mentioned earlier, occupy a prominent place among the articles. Hydrolysis is the word with the lowest number among these, as it is still developing research using lignocellulosic materials and may be an emerging trend in the future of scientific literature.

#### 3.4.2. Research Hotspots

We use CiteSpace software (Chaomei Chen of Drexel University, Philadelphia, PA, USA) which acts on the database analysis to delineate which trends stand out and thus define which ones emerge in the current research topic. CiteSpace is used to facilitate the analysis of the intellectual structure and emerging trends [157], as well as to improve the understanding of platform research and enable future developments for theorists and practitioners [158].

**Figure 17:**
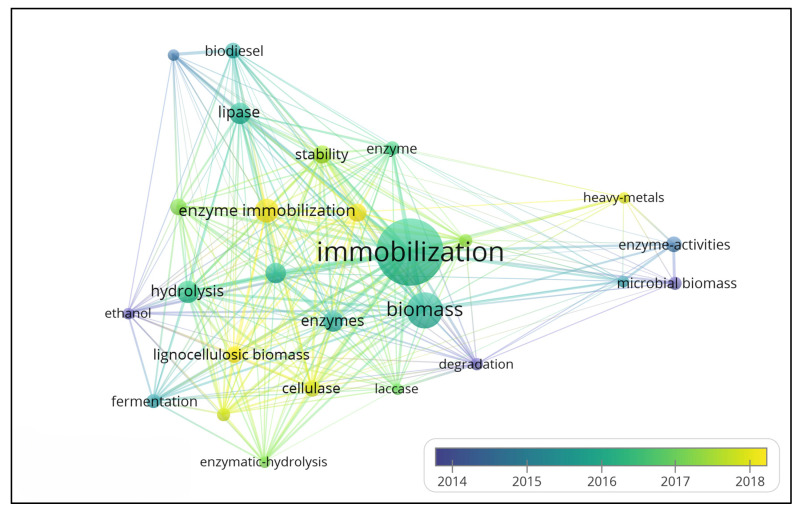
The relevant keyword usage evolution map over time.

**Figure 18:**
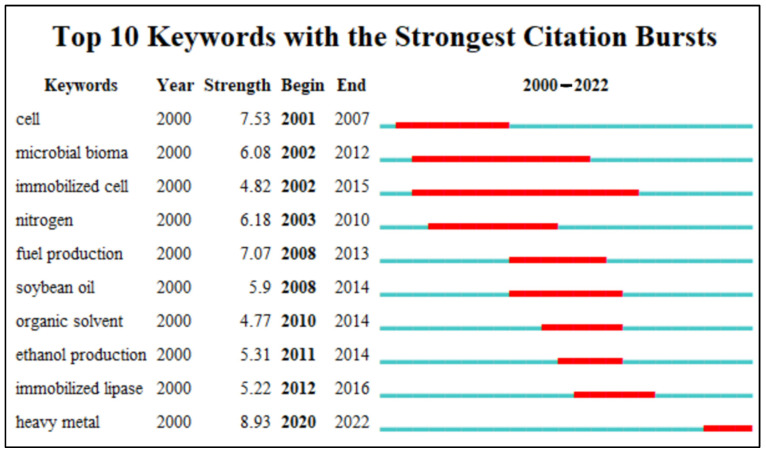
List of high-impact keywords from 2000 to 2022.

In the visualization of Figure 17, it is clearly shown that the three main categories of subjects are Immobilization, Biomass, and Enzymes. In recent years, as can be seen in Figure 18, the four keywords with the highest intensity of emergence are heave metal (8.93), cell (7.53), fuel production (7.07), and nitrogen (6.18). In addition, the keywords Ethanol Production and Immobilized Lipase during 2011–2014 and 2012–2016, respectively, indicate that this area of research has become very prevalent in recent years, and it can be assumed that research on these topics has been encouraged and widely developed. The words involving immobilization and enzymes reinforce the importance of this area of research and, consequently, strengthen the need for new materials for study and application.

##### Constant Fields of Investigation

The finding of the increase in the relevance of an area is directly related to the growth in the number of publications that this area receives. Thus, the keywords will be used as guides todetermine the path of the following trends of future studies that will be linked to using lignocellulosic materials for enzyme immobilization. Table 5 presents the top six sets of co-citations among the manuscripts present in the study database that will refer to the same subject.

Cluster #0 has the keyword “Microbial activity”, which, like the other words, refers to microorganisms. Microbial biomass is one of the potential materials used as enzyme support and fits within lignocellulosic materials [12]. The article “Role of biochar and *Eisenia fetida* on metal bioavailability and biochar effects on earthworm fitness” is one of the representatives of this cluster and conducts studies on the individual and combined effects of biochar and earthworms (*Eisenia fetida*) on soil properties, bioavailability, and earthworm fitness in soils historically contaminated with heavy metals [159]. Cluster #5 is closely related to this cluster.

The keyword “Enzyme hydrolysis” represents cluster #1 and, together with the other highlighted words, defines this group as the representative enzyme immobilization. Enzymes can be used in the synthesis reactions of many products; thus, different ways to improve these enzymes are studied, such as improving thermal stability (as highlighted in the cluster), selectivity, and resistance to denaturation [78]. Here, the immobilization is on lignocellulosic material, but the closest can be immobilized on mesoporous silica [170]. The manuscript “Novel Magnetic Cross-Linked cellulase Aggregates with a Potential Application in Lignocellulosic Biomass Bioconversion” is one representative of this cluster and explains that the stabilization technique can improve the stability and recyclability of enzymes and thus a novel cross-linked cellulase magnetic complex was developed and applied to biomass biotransformation [13].

Cluster #2 is headed by the keyword “White-rot fungi”, which is directly related to the other highlighted words. White-rot fungi can synthesize enzymes and are related to the topic of this paper. An example of an enzyme obtained from white-rot fungi is the enzyme lignin peroxidase, obtained from the fungus *Phanerochaete chrysosporium* [171]. This cluster is represented by the manuscript “Green production of a yellow lacase by *Coriolopsis gallica* for phenolic pollutants Removal” which addresses research on a new effective green production strategy for lacase fungi and showed that the resulting purified lacase from *C. gallica* showed good enzymatic properties and catalytic potential for phenolic pollutants’ removal [162].

Cluster #3 has the keyword “Chromatographic behavior” as a representative. In this cluster, the multidisciplinarity of the theme of this work was evident, since this cluster presents from lignocellulosic materials, such as tomato pomace, to examples of chemical analyses performed, such as chromatography. The article “Sparge gas composition affects biomass and ajmalicine production from immobilized cell cultures of *Catharanthus roseus*” represents this cluster and addresses the effects of O_2_ and CO_2_ on secondary development and metabolism that were investigated using ajmaline production from *Catharanthus roseus* cultures [164].

Cluster #4 is represented by the keyword “Biodiesel Production”. This word represents this cluster well, as all the highlighted words are related to biodiesel production. This area is another possibility for the application of enzymes, especially immobilized enzymes. Here, we can already see that the environmental issue is being emphasized in the theme of this study because some alternative biofuels to petroleum derivatives have already been cited. The manuscript “Enzymatic production of biodiesel from microalgal oil using ethyl acetate as an acyl acceptor” is the highlight of this cluster and presents a study on the application of ethyl acetate in the enzymatic production of biodiesel, in a pioneering way, using the microalgae *Chlorella vulgaris*, as a source of triglycerides. In this manuscript, the enzymatic conversion of fatty acids to biodiesel was catalyzed by Novozym 435, which is an efficiently immobilized lipase for biodiesel production [166].

## 4. Future Trends

Future prospects in this area are:

- Increase and strengthening of cooperation between emerging countries to enhance research with lignocellulosic materials;
- Strengthening of relations between organizations with researchers that follow the same objective, guaranteeing an exchange of data and information that will be significant for the development of this area, where enzymatic immobilization in lignocellulosic materials is carried out;
- The highlighted keywords analyzed showed the future trend for this area, where these new biomaterials will be applied in the most diverse research;
- Finally, an increase in the applicability of these materials is expected in order to reduce the impacts involved in incorrect disposal in the environment.

## 5. Conclusions

With the advanced bibliometric analysis, it is possible to highlight that the application of lignocellulosic materials as enzyme support has, as its focus, concerns with environmental problems caused by production lines that use the chemical route. In addition, many methodologies aim to circumvent such mishaps to ensure a more sustainable market for lignocellulosic materials.

With regard to the origin of the periodicals that address this theme, the large role of European periodicals was evident. A large number of journals leads to the conclusion that there are many application possibilities for the theme in question, as each journal has its own aims and scopes. Based on this data, an analysis of the countries that host these journals was carried out, which leads to the understanding that some countries stand out to the detriment of others. We believe that this is due to the economic scenario of these countries. Countries such as the USA, China, India, and Brazil are frequently cited in the analyses. These countries have well-developed economies or stand out among developing economies in common.

Concerning the numbers, Chinese prominence is unquestionable. China produces 28.07% of the articles on lignocellulosic materials for enzyme immobilization. This achievement guarantees it a high-impact position in this area and shows its prospects in this research line. In addition, China has the highest percentage of citations among all countries in the database.

Another interesting point concerns the collaboration that exists between countries. From the analyses performed, it is possible to conclude that cooperation exists within each country—being carried out between internal organizations—with promising results regarding academic production. It also exists between countries, where borders are no longer barriers and become bridges for exchanging information and knowledge. This exchange of information, research, and technology is essential for developing countries, research areas, and the topics in focus on lignocellulosic materials as enzymatic support.

The lignocellulosic biomass as a support for enzyme immobilization is an alternative to be considered because it composes a sustainable method and meets problems related to storing and handling of these residues. The immobilization in lignocellulosic materials has been highlighted in scientific research in several areas, such as chemical, pharmaceutical, biotechnological, food, and environmental industries. Therefore, it is possible to determine that biocatalysts on lignocellulosic supports represent an environmentally friendly method and aim to make bio-processes in this area more sustainable.

## Figures and Tables

**Table 1:** Top 10 scientific journals published on the use of lignocellulosic residues as supports for enzyme immobilization, ranked by the number of publications.

Rank	Journal	Publisher	Country	Number of Publications	Impact Factor	Number of Citations	Average Citations	Percentage (%)
1	Bioresource Technology	Elsevier	ENG	51	11.889	2913	57.12	4.21
2	Applied Biochemistry and Biotechnology	Springer	USA	50	3.094	1774	14.94	4.13
3	Enzyme and Microbial Technology	Elsevier	USA	37	3.705	1196	32.32	3.06
4	Biomass and Bioenergy	Elsevier	ENG	24	5.774	701	29.21	1.98
5	Chemosphere	Elsevier	ENG	23	8.943	368	16.00	1.90
6	Process Biochemistry	Elsevier	ENG	23	4.885	1025	44.57	1.90
7	Science of The Total Environment	Elsevier	NLD	21	10.754	224	10.67	1.73
8	Waste and Biomass Valorization	Springer	NLD	21	3.449	699	33.29	1.73
9	Soil Biology and Biochemistry	Elsevier	ENG	20	8.546	1620	81.00	1.65
10	International Journal of Biological Macromolecules	Elsevier	NLD	18	8.025	250	13.89	1.49

USA = United States of America; NLD = Netherlands; ENG = England.

**Table 2:** The 10 most productive countries on the use of lignocellulosic residues as supports for enzyme immobilization.

Rank	Country	Number of Publications	Number of Citations	Average Citation	Total Link Strength	Percentage (%)
1	China	340	7419	21.82	57.67	28.07
2	India	163	5399	33.12	37.78	13.46
3	United States of America	126	4126	32.74	23.93	10.4
4	Brazil	111	1598	14.39	28.46	9.16
5	South Korea	68	2462	36.20	14.94	5.61
6	Germany	60	2947	49.11	11.95	4.95
7	Spain	58	1191	20.53	14.24	4.78
8	Italy	54	1184	21.92	9.79	4.45
9	Japan	43	2305	53.60	7.80	3.55
10	Australia	41	1044	25.46	12.72	3.38

**Table 3:** The most cited papers on the use of lignocellulosic residues as supports for enzymatic immobilization.

Rank	Paper	Authors	Year of Publication	Annual Average Citations	Total Citations
1	Ethanol fermentation from biomass resources: current state and prospects	Lin, Y, and Tanaka, S	2006	59.88	1018
2	Fermentation of lignocellulosic hydrolysates. I: inhibition and detoxification	Palmqvist, E and Hahn -Hagerdal, B	2000	40.83	939
3	Fungus-mediated synthesis of silver nanoparticles and their immobilization in the mycelial matrix: A novel biological approach to nanoparticle synthesis	Mukherjee, P; Ahmad, A; Mandal, D; Senapati, S; Sainkar, SR; Khan, MI; Parishcha, R; Ajaykumar, PV; Alam, M; Kumar, R, and Sastry, M	2001	38.41	845
4	White-rot fungi and their enzymes for the treatment of industrial dye effluents	Wesenberg, D; Kyriakides, I and Agathos, SN	2003	38.9	778
5	An overview of enzymatic production of biodiesel	Ranganathan, SV; Narasimhan, SL and Muthukumar, K	2008	29.8	447
6	Fungal dye decolorization: Recent advances and future potential	Kaushik, P and Malik, A	2009	28.71	402
7	Pathways of nitrogen utilization by soil microorganisms—A review	Geisseler, D; Horwath, WR; Joergensen, RG and Ludwig, B	2010	29.46	383
8	Production of secondary metabolites from cell and organ cultures: strategies and approaches for biomass improvement and metabolite accumulation	Murthy, HN; Lee, EJ, and Paek, KY	2014	36.78	331
9	Enzyme stabilization by nano/ microsized hybrid materials	Hwang, ET and Gu, MB	2013	30.6	306
10	The role of additives on anaerobic digestion: A review	Romero- Guiza, MS; Vila, JJ; Mata-Alvarez, J; Chimenos, JM and Astals, S	2016	43.43	304

**Table 4:** The twenty most prominent keywords in article search.

Rank	Keyword	Frequency	Total Link Strength	Rank	Keyword	Frequency	Total Link Strength
1	immobilization	385	622	11	Lignocellulosic biomass	80	170
2	Biomass	192	290	12	optimization	80	169
3	Enzyme immobilization	125	234	13	Biodiesel	78	108
4	hydrolysis	116	262	14	Enzyme-activities	76	64
5	lipase	107	220	15	fermentation	69	126
6	purification	106	225	16	Enzyme	67	130
7	enzymes	106	184	17	adsorption	66	123
8	nanoparticles	91	191	18	pretreatment	65	160
9	stability	89	236	19	Microbial biomass	64	54
10	cellulase	80	237	20	degradation	62	93

**Table 5:** The top six feedstock co-citation clusters on the use of lignocellulosic residues as supports for enzyme immobilization, based on the CiteSpace analysis.

Cluster ID	Label	Node Size	Mean	Top Five Terms	Representative Articles
#0	Microbial activity	159	2010	Microbial activity; agricultural soil; microbial biomass; soil microorganism; temperate forest soil	[159,160]
#1	Enzymatic hydrolysis	145	2014	Enzymatic hydrolysis; lignocellulosic biomass; situ saccharifications; thermal stability; mesoporous silica	[13,161]
#2	white rot fungi	63	2009	White rot fungi; ligninolytic enzyme; *Phanerochaete chrysosporium*; white rot; dye decolorization	[162,163]
#3	Chromatographic behavior	55	2006	Chromatographic behavior; chelate metal; continuous production; tomato pomace; hydrophilic polyurethane foam	[164]
#4	Biodiesel production	53	2012	Biodiesel production; enzymatic production; solvent-free system; recombinant *Rhizopus oryzae* lipase; biotechnological production	[165,166]
#5	microbial growth	52	2008	Microbial growth; yarrow lipolytic; biobased production; lignocellulosic hydrolysate; lip2 lipase	[10,167]
#6	semiarid area	34	2006	semiarid area; process engineering aspect; *Acremonium chrysogenum*; complex media; *Cephalosporin c*	[168,169]

## Data Availability

Not applicable.
